# Activation of Nm23-H1 to suppress breast cancer metastasis via redox regulation

**DOI:** 10.1038/s12276-021-00575-1

**Published:** 2021-03-22

**Authors:** Bokyung Kim, Kong-Joo Lee

**Affiliations:** College of Pharmacy and Graduate School of Pharmaceutical Sciences, Ewha Womans University, Seoul, South Korea

**Keywords:** Breast cancer, Metastasis, Drug development

## Abstract

Non-metastatic protein 23 H1 (Nm23-H1), a housekeeping enzyme, is a nucleoside diphosphate kinase-A (NDPK-A). It was the first identified metastasis suppressor protein. Nm23-H1 prolongs disease-free survival and is associated with a good prognosis in breast cancer patients. However, the molecular mechanisms underlying the role of Nm23-H1 in biological processes are still not well understood. This is a review of recent studies focusing on controlling NDPK activity based on the redox regulation of Nm23-H1, structural, and functional changes associated with the oxidation of cysteine residues, and the relationship between NDPK activity and cancer metastasis. Further understanding of the redox regulation of the NDPK function will likely provide a new perspective for developing new strategies for the activation of NDPK-A in suppressing cancer metastasis.

## Breast cancer: stopping the spread

A small molecule called NMac1 from a plant related to ginger boosts the activity of Nm23-H1, a protein-suppressing metastasis, the spread of cancer cells to other tissues. Metastasis is a primary cause of death in cancer patients. Efforts to develop remedies based on Nm23-H1 treatments have had limited success. Kong-Joo Lee and Bokyung Kim at Ewha Womans University in Seoul, South Korea, reviewed discoveries about the molecular basis of Nm23-H1 action. They report that Nm23-H1 is easily oxidized in cells, preventing it from performing its anti-metastasis function. However, the small molecule NMac1 can stabilize Nm23-H1 by preventing oxidation, and action of NMac1 on Nm23-H1 was shown to prevent breast cancer metastasis in mice. This improved understanding of the molecular mechanism is the first step to potential new anti-metastasis treatments.

## Introduction

Metastasis, a major cause of death in cancer patients, is a complex process that includes invasion of the primary tumor, intravasation into blood vessels, circulation under anoikis resistance, extravasation, and colonization at distant sites. However, as our current understanding of the clinical, translational, molecular, and biochemical mechanisms underlying sequential metastatic processes is insufficient for designing rational approaches to prevent or arrest metastatic processes, strategies for suppressing metastasis have met with little success.

To control metastasis, a number of metastasis suppressor proteins (MSPs) have been identified and studied in human carcinomas, where they mostly act by altering metastasis-related signal transduction. The first discovered and best-known MSP is Nm23 (non-metastatic clone 23), which inhibits kinase activity that facilitates cell division in melanoma, breast cancer, and colon cancers^1^. Mitogen-activated protein kinase kinase 4 (MKK4) and mitogen-activated kinase 7 (MAK7) are also MSPs that suppress metastasis by affecting the mitogen-activated protein kinase (MAPK) pathway^2^. Kangai 1 (KAI1/CD82) is another human MSP identified in prostate cancer cells, and the expression of KAI1 in prostate cancer cells was shown to inhibit the progression of lung cancer^3–5^. On the other hand, KAI1 has recently been investigated for its regulatory function that maintains the dormancy of long-term hematopoietic stem cells by interacting with DARC^6^. Kisspeptin 1 (KISS1) is also a well-characterized MSP that is frequently lost in metastatic melanoma and can suppress metastasis in these cells^7^. Bone morphogenic protein 4 (BMP4) is a secreted factor that belongs to the TGF-β superfamily of proteins. Reduced BMP4 expression was associated with increased metastatic potential in mouse mammary tumors^8^. A number of other MSPs, including various TIMPs, gelsolin, cadherins, DRG1, and TXNIP, have also been shown to suppress metastasis via various regulatory pathways^9^. Among these MSPs, Nm23-H1, also called nucleoside diphosphate kinase-A (NDPK-A) and NME1, has been the object of intense focus in various cancers^10,11^. Nm23-H1 suppresses metastasis by inhibiting multiple metastatic steps, including invasion of the primary site and colonization^12^. The molecular mechanism of the anti-metastatic potential of Nm23-H1 was elucidated in the cytoskeleton-organizing pathway and in the MAPK signaling pathway based on its physical interaction with kinase suppressors of Ras-1/2 (KSR-1/2)^13^.

Nm23 is in a family of histidine kinases with NDPK activity that catalyzes the transfer of high-energy γ-phosphate from nucleoside triphosphate (NTP) to nucleoside diphosphate (NDP) in a reversible manner to maintain the equilibrium of NTPs inside the cell^14^. In addition, Nm23-H1 possesses multiple enzymatic activities, including 3′–5′ exonuclease activity. Among these functions, NDPK activity is crucial for Nm23-H1-mediated biological functions, including cytoskeletal organization, insulin secretion, and endocytosis^12,13,15^.

The Nm23 family in human consists of ten NME (non-metastatic enzyme) protein members (from Nm23-H1 to Nm23-H10 and from NME1 to NME10) identified thus far, which are classified into two groups according to enzyme activity and sequence homology. Group 1 isoforms NME1~4 have highly homologous sequences with NDPK activity, whereas Group 2 isoforms NME5~10 have divergent sequences showing no or low NDPK activity^15^. In Group 1 NMEs, Nm23-H4 has a specific mitochondrial targeting sequence in the N-terminus, providing GTP to OPA1 (optic atrophy 1), a dynamin-related GTPase in mitochondria. It also contributes to cardiolipin transfer in the intermembrane space and participates in mitochondrial respiration by providing ADP^16^. Recently, Nm23-H3 was shown to contribute to mitochondrial fission by providing GTP as fuel for Drp1 at the mitochondrial outer membrane, but further research is needed^17^.

As Nm23 has NDPK activity, which means it has high affinity for NTPs, Nm23-H1 and Nm23-H2 can be purified using ATP-sepharose affinity column chromatography because Nm23 has the highest affinity for ATP among the proteins in cell lysates^18^. Nm23-H1 and Nm23-H2 are the most abundant Nm23 isoforms in human cells and are also known to suppress metastasis in multiple tumor types^19^. Loss of Nm23-H1 expression was shown to correlate with the degree of metastasis and prognosis in breast, ovarian, melanoma, gastric, and lung carcinomas^20–24^. It has been suggested that Nm23-H2, although less involved than Nm23-H1, suppresses tumor metastasis by influencing the expression of cell adhesion molecules such as vinculin, plakoglobin and their organization^25,26^. Intriguingly, Nm23-H1 and Nm23-H2 have 88% identical sequences and similar enzymatic activities. Their N- and C-termini are both highly conserved, as are the Kpn loop and active site (H118) region^27^. However, they have different stabilities and biological functions, and regulate different signaling pathways by interacting with distinct partner proteins, as shown in Table 1. As the understanding of the differences between Nm23-H1 and Nm23-H2 at the molecular level is insufficient, our group focused on recent findings to explain the changes in structure and function based on oxidative regulation.

**Table 1:** Comparison of the biochemical and cellular properties of Nm23-H1 and Nm23-H2.

	Nm23-H1 (NME1)	Nm23-H2 (NME2)
Sequence N- and C-termini (Bold: Kpn loop aa 94–114, Active site H118)	H1 ^1^mancertfia^10 94^**tnpadskpgtirgdfci qvgr**nii**h**gs^120 140^dytsca^146^ H2 ^1^manlertfia^10 94^**tnpadskpgtirgdfci qvgr**nii**h**gs^120 140^dyksca^146^	
Biological activities	NDP kinase activity & histidine kinase activity (active site H118) metastasis suppressor activity (NME1>NME2)	
	3′–5′ exonuclease activity	Transactivation activity on *c-myc*
Cancer relationship	Highly related in cancer metastasis suppression and prognosis of cancer patients	Less involvement in suppression of cancer metastasis
Stability^30^	Unstable (easily oxidized)	Very stable
Cellular location	Cytoplasm	Cytoplasm and nucleus
Active structure	Homo- or hetero-hexamer structure (in mammals)	
Interacting proteins^35^	Dynamin^91^	
	KSR-1/2^13^, CKI^28^, Aurora-A/STK15^92^, EBNA-1& EBNA-3C^31^, Rad^33^, Tiam1^34^, Dbl-1^32^, h-Prune^93^, MIF^94^, p53/STRAP^95^, VHL^96^, CDC42^97^ and other small GTPase-interacting proteins^37^	TRF1^98^, MDM2^99^, ICAP1α^100^ etc.

NDPK activity has been widely found to be affected by phosphorylation. It also affects cell motility since the phosphorylation state of Nm23-H1 regulates its interaction with phosphatase h-Prune^28^. Casein kinase I (CKI) phosphorylates the Ser120, 122, and 125 residues of Nm23-H1 and increases its interaction with h-Prune. As a result of Nm23-H1-h-Prune complex formation, the NDPK activity of Nm23-H1 is inhibited, and the PDE activity of h-Prune is activated^28^. In addition, Aurora-A/STK15 can phosphorylate Nm23-H1, but no specific phosphorylation or enzymatic alteration is involved^92^. However, we found that Nm23-H1 easily loses its NDPK enzymatic activity when not phosphorylated in vitro and that Cys109 in Nm23-H1 is easily oxidized to various oxidation states, including intra- and inter-disulfide cross-links, glutathionylation, and sulfonic acid formation. Mutation of Cys109 to Ala (C109A) in Nm23-H1 stabilizes its NDPK enzymatic activity and promotes its anti-metastatic potential, which suggests that the key enzymatic and metastasis suppressor functions of Nm23-H1 are regulated by the oxidoreduction of its Cys109 residue^29^. In addition, the hexameric state of Nm23-H1, which is required to suppress metastasis, shows significant structural changes and dissociates into dimers upon oxidation^30^. We specifically highlight the relationship between the oxidation states of Nm23-H1 and its enzymatic activity, as well as structural changes, and make various attempts with novel strategies to inhibit metastasis by activating NDPK-A.

Regarding downstream effectors of Nm23-H1, EBNA-1 and EBNA-3C interact with Nm23-H1 and induce its translocation to the nucleus^31^. Nm23-H1 also interacts with and modulates GTP-binding proteins such as Ras-related GTPase (Rad), T-cell lymphoma invasion and metastasis-inducing protein 1 (Tiam1) and BMP-like-1 (Dbl-1). Through interaction with Rad, Nm23-H1 regulates the Rad-GTP to Rad-GDP ratio and affects downstream effector proteins involved in cytoskeletal organization and cell motility. The interactions with Tiam1 and Dbl-1 also regulate guanine exchange factor (GEF) activity^32–34^.

## Nm23-H1 as a metastasis suppressor

### Nm23-H1 is a key metastasis suppressor in breast cancer and melanoma

Nm23-H1 was first identified as a metastasis suppressor by Steeg et al.^36^. Nm23 expression in highly metastatic K1745 murine melanoma cells was lower than that in poorly metastatic clones, as determined by differential hybridization. Since this discovery, the clinical relevance of Nm23-H1 has been extensively studied in tumor metastasis in an in vivo mouse xenograft model and clinical samples and in cell invasion in various in vitro cell lines. The results from many studies are controversial and depend on the type of cancer studied. NDPKs are known to regulate the amount of GTP available for G-protein activation in vitro, but they were not identified in vivo. Nm23-H1 directly interacts with small G proteins such as Rac1 and Cdc42, which are facilitators of cell migration, and simultaneously suppresses GEFs or acts as a GTPase-activating protein (GAP) for G-protein-related signal transduction^37,38^.

A deeper understanding of Nm23-H1 is needed to inhibit metastasis in the cancer microenvironment where small G proteins such as Rac1 are upregulated. Rac1 was recently reported to be overexpressed in proliferative breast disease^39^. In addition to the overexpression of Rac1, low expression of Nm23-H1 is observed in aggressive breast cancer, including triple-negative breast cancer subtype (TNBC). This suggests that the activity of the overexpressed G-protein can be reduced, leading to a reduction in breast cancer metastasis by regulating NM23-H1.

The inverse relationship between Nm23-H1 expression and metastatic potential is well-characterized in breast cancer. This inverse relationship was also demonstrated with in vivo xenografts and in vitro models of melanoma, breast, lung, liver, ovarian, and several other aggressive carcinomas^12,39–47^. However, the overexpression of Nm23-H1 led to poor prognosis in several cancers, including hematological malignancies, neuroblastoma, ovarian cancers, and prostate cancers^47,48,50,51^. A S120G missense mutation was identified in several aggressive cases of neuroblastomas, and seems to be specific to this tumor type, and led to the loss of Nm23-H1 functions that subsequently cause poor prognosis when Nm23-H1 is overexpressed^48^. Nevertheless, there have still been many reports, suggesting that Nm23-H1 overexpression inhibits metastasis and leads to better overall survival or prognosis in many aggressive cancers, as mentioned above. Thus, the question of whether activating or upregulating Nm23-H1 inhibits metastasis in aggressive cancers remains unanswered but is an attractive possibility. Based on the clinical evidence of an inverse relationship, there have been constitutive attempts to inhibit metastasis by upregulating or activating Nm23-H1.

### Attempts to increase the cellular level of Nm23-H1 to augment the suppression of tumor metastasis

As Nm23-H1 is inversely correlated with cancer metastasis, it is reasonable to hypothesize that increasing Nm23-H1 levels might suppress metastasis. Many studies were conducted to test the hypothesis. First, gene therapy approaches were attempted using a viral vector of Nm23. This approach, involving transferring adeno-associated virus (AAV)-mediated genes, increased the exogenous gene expression of Nm23-H1 by >95% in an orthotropic implantation model of ovarian cancer. A significant reduction in liver metastasis and an increase in median survival were also observed, but the clinical usefulness of these findings was limited because of possible vector-related toxicity and short-term expression of the transgene^47^.

Second, indirect approaches were attempted to promote Nm23-H1 expression by treating cells and tissues with various compounds. Several studies found that Nm23-H1 expression is increased by anti-inflammatory agents, including indomethacin (a cyclooxygenase inhibitor), acetylsalicylic acid, l-carnosine, γ-linoleic acid, and glucocorticoids, whereas proinflammatory molecules, including prostaglandin E2 (PGE2), TNF-α, and IFN-γ, reduced Nm23 expression in B16F10 murine melanoma cells^52–54^. As Nm23 expression is regulated by glucocorticoids, medroxyprogesterone acetate (MPA), which is an agonist of the glucocorticoid receptor, was employed to increase the transcription of Nm23-H1 both in vivo and in vitro^55,56^. MPA induced a significant increase in Nm23-H1 expression, suppressing metastasis in mouse breast cancer model systems in 2003^56^. MPA also successfully induced Nm23-H1 expression in TNBC in vitro and decreased pulmonary metastases by ~43% in xenograft models. Based on this animal study, phase 2 clinical studies were carried out using MPA at 1,000–1,500 mg/day orally alone or in combination with low-dose chemotherapy in 30 postmenopausal women with hormone receptor-negative breast cancer. However, the efficacy of MPA was not clinically sufficient, possibly because of its poor bioavailability^57^.

Third, protein therapy was attempted in which direct transduction of cell-permeable Nm23-H1 protein (CP-Nm23-H1) was assessed and found to decrease metastasis^49^. His-tagged CP-Nm23-H1 was generated and introduced systemically in a xenograft model of metastatic breast cancer. Increased CP-Nm23 expression in MDA-MB-435 breast cancer cells inhibited pulmonary metastases and prolonged the survival of tumor-bearing animals. These results confirmed that CP-Nm23-H1 can be used as adjuvant therapy against disseminated cancers. Compared with the indirect induction of Nm23-H1 expression with MPA, the direct introduction of CP-NM23-H1 presented better efficacy in metastasis suppression and increased animal survival. However, proteins have limitations when used as therapeutics because of the nonspecific effects of extracellular Nm23-H1 and undesirable widespread entry of Nm23-H1 into non-targeted cells, which are considered concerning issues.

### Molecular mechanism underlying the oxidative regulation of Nm23-H1 in cells

As no methodology is available to increase Nm23-H1 expression or NDPK-A activity in an approach to control metastasis, we first tried to understand the biochemical and molecular regulatory mechanism of Nm23-H1 activity based on reactive oxygen species (ROS)-controlled enzyme activity. Active Nm23-H1 is a hexamer, and the stability of its NDPK activity is low compared with that of Nm23-H2, even though these two isoforms have very similar sequences and crystal structure^30^.

#### Redox regulation of proteins in response to ROS in cancer

Reactive oxygen species (ROS), which are necessary for cellular homeostasis but fatal to cells when their cellular level is out of an adequate level, are balanced by regulated production and elimination. ROS generated by external stimuli, growth factors, cytokines, metabolites, and infections act as signaling molecules for the oxidation of target proteins and for the regulation of on–off switch proteins that participate in signaling pathways. Enzymes containing Cys residues in the active site, including phosphatases, kinases, oxidoreductases, and cysteine proteases, and proteins containing redox-sensitive Cys residues, regulate various cellular processes such as cell proliferation, differentiation, migration; metastasis/angiogenesis; inflammation; and death^58–61^. Many studies have corroborated roles of ROS in cancer metastasis and angiogenesis^61–63^. ROS promote cancer initiation by accelerating mutagenesis and modulating signaling pathways related to proliferation, survival, and stress resistance^64^. Increased intracellular ROS induce MAPK activation and consequently regulate cell migration, tubule formation and angiogenesis both in vitro and in vivo^61^. Elevated mitochondrial ROS also activate MAPK cascades and enhance the metastasis of breast and colon cancer cells, such as 4T1, HCT116, and HT29 cells^65^. Ubiquitin carboxy-terminal hydrolase L1 (UCH-L1) protein deubiquitinates and upregulates NOX4 to generate H_2_O_2_ and the overexpression of UCH-L1 promoted the invasive potential of the B16F10 lung cancer cell line via regulating the upstream kinase Akt, suggesting that ROS are related to cancer aggressiveness in various pathways^66^.

Most cellular ROS originate from superoxide anion (O_2_^•−^), which are readily converted to H_2_O_2_ by the superoxide dismutase (SOD) family of enzymes and then reduced to H_2_O by catalase, glutathione peroxidase, or peroxiredoxin (PRDX or Prx) families of enzymes^60^. These types of cellular ROS primarily oxidize Cys residues in proteins. The Cys residue, the least abundant among the amino acids (1~2%), seems to have unique features that enable it to significantly influence protein structure and function and its enzymatic activity^67^. Redox-sensitive Cys is believed to have a relatively low pK_a_^68^ and is one of the major targets of ROS in biological systems^69^ since the thiolate form (Cys-S^−^) of Cys is required for oxidation by ROS. Only a limited number of proteins that are sensitive to oxidation/reduction processes have been described: PTEN phosphatase; protein tyrosine phosphatase; PI3-kinase; MAPK; ROS homeostasis-related proteins; proteins encoded by antioxidant genes that regulate thioredoxin (Trx), Prx, Ref-1 and Nrf2; mitochondrial oxidative stress-related proteins; apoptosis-related p66Shc; and ATM-regulated DNA damage proteins^60,70^. In addition, Hsp33 is reported to have redox-sensitive cysteines, whose oxidation status controls its ability to unfold proteins as part of its chaperone function^71–73^. A recent study employing a novel chemical probe for specifically capturing reactive Cys residues, called NPSB-B or Ctag, combined with proteomics was used to identify a variety of ROS target proteins (226 proteins) having redox-sensitive Cys sulfhydryls^74^. The results show that the identified redox-sensitive proteins include known redox-sensitive proteins and many novel proteins having diverse redox sensitivity.

In nonphagocytic cells, relatively low levels of ROS are continuously produced by NADPH oxidase (NOX), which is activated by various growth factors (e.g., EGF, PDGF, and VEGF) and cytokines (e.g., TNF-α and IL-1), by oxidative phosphorylation in mitochondria or by oxidative protein folding in the ER^60,77,78^. Oxidative changes in ROS target proteins in specific sites and modification species, their molecular activities and structures, changes in the interactome, and the effects of ROS on various signaling pathways are reversible because of antioxidant systems, including the Trx and Grx systems. Nm23-H1 is also a ROS target protein with another type of regulatory mechanism. As quaternary structural changes from hexamers to dimers occur by oxidative stress, the molecular mechanism for the oxidative regulation of Nm23-H1 was identified via biochemical and cellular studies combining X-ray crystallography and hydrogen-deuterium exchange mass spectrometry (HDX-MS)^29,30,74–76,102,103^.

#### Oxidoreduction of Nm23-H1

The cellular NDPK activity of Nm23-H1 is tightly regulated, readily inactivated by low levels of ROS generated by external stimuli, including growth factors, cytokines, and various stresses, and reactivated by the NADPH-TrxR-Trx oxidoreduction system^29^. On the other hand, further stepwise oxidation triggers a large conformational change that induces Cys109 oxidation to sulfonic acid or glutathionylation and the irreversible loss of NDPK activity.

The NDPK activity of Nm23-H1, but not that of Nm23-H2, easily disappears upon the oxidation of Cys109 to sulfonic acid, even though the regions containing Cys109 and active site H118 in both isoforms are 100% identical^29,103^ (Table 1). Nm23-H1 was therefore presumed to be a target of ROS. Upon oxidation, Nm23-H1 hexamers dissociate into dimers, by which it loses its metastatic potential and NDPK activity in invasive MDA-MB-231 cells. The C109A mutant is constitutively active as an NDPK and metastasis suppressor. Among the three Cys residues of Nm23-H1, Cys4 is not conserved in mammals (<15%) nor in Nm23-H2 (Table 1). To resolve the enigma of Nm23-H1 redox regulation and its relationship of conserved Cys, the NDPK activity of all three Cys mutants (Cys4, 109, and 145) was examined, and the activity of all three mutants was conserved even under oxidative stress, which indicates that all three Cys residues play roles in redox regulation. Under nonreducing conditions with oxidative stress, an intramolecular disulfide bond between Cys4 and Cys145 was detected using MS/MS combining the disulfide bond-searching algorithm DBond^79^. Employing the chemical probe NPSB-B (Ctag), which can specifically label redox-sensitive Cys residues^74^, Cys4 was confirmed to be a peroxidatic Cys or reactive Cys-S^−^ that forms a disulfide bond with resolving Cys145. The effect of Cys4–Cys145 intra-disulfide cross-linking on the oxidation of Cys109 to sulfonic acid was assessed by measuring the degree of oxidation to sulfonic acid with peptide sequencing with nanoUPLC-ESI-q-TOF tandem MS. Intriguingly, Cys109 in Nm23-H1 wild type was heavily oxidized to sulfonic acid in a ROS-dependent manner, whereas it was oxidized to a much lower degree in the C4S and C145S mutants. These findings demonstrate that Cys4–Cys145 intra-disulfide cross-linking is a prerequisite for changing the conformation of Nm23-H1 and inducing the oxidation of Cys109 to sulfonic acid and glutathionylation.

Reversible S-glutathionylation is known as an important post-translational modification, protecting protein cysteines from irreversible oxidation and serving to transduce redox signals. For example, p53 is inhibited by glutathionylation at cysteine residues in the proximal DNA-binding domain during oxidative stress and reversed by the redox system^80,81^. These findings suggest that Cys109 has a key role in the cellular response to oxidative stress via glutathionylation and disulfide formation. This study was the first to show the molecular regulation of enzymes through stepwise oxidative modification. In addition, it may explain why Nm23-H2, lacking Cys4, is stably active under oxidative stresses.

To investigate whether the Cys4–Cys145 intra-disulfide bond is biologically regulated, proteins that interact with oxidized Nm23-H1 were identified. Oxidized Nm23-H1 lost its enzymatic activity and its ability to suppress tumor metastasis, specifically interacts with thioredoxin reductase 1 (TrxR1), which is known as a selenium-containing pyridine nucleotide disulfide oxidoreductase catalyzing NADPH-dependent reduction of Trx^29^. The NDPK activity of oxidized Nm23-H1 is recovered by reducing the Cys4–Cys145 intra-disulfide bond to free sulfhydryls through the NADPH-TrxR-Trx system; this system can reduce oxidized disulfide bonds on proteins by interacting with the redox-active center of Trx (CGPC, Trx-(SH)_2_) to form a disulfide bond (Trx-S_2_) that can be sequentially reduced by TrxR and NADPH. As a major reducing system, NADPH-TrxR-Trx maintains the cellular redox balance through the reversible reduction of various proteins oxidized by ROS.

#### Structural analysis of oxidized Nm23-H1

To understand the molecular mechanisms underlying the stepwise oxidation to the Cys4–Cys145 disulfide bond, leading to Cys109 oxidation and to identify how oligomeric states are changed from hexamers to dimers during oxidation, dynamic structural changes were examined with HDX-MS, and conformational changes of oxidized Nm23-H1 were explored by X-ray crystallography.

X-ray crystal structures are available for Nm23-H1 in the PDB (the Protein Data Bank), including those of native^75,103^, single-mutant^82^, and double-mutant^83^ forms. All forms of Nm23-H1 exist as hexamers with D3 symmetry, and each subunit of the hexamer has a globular alpha/beta domain with a ferredoxin-like fold and an extended C-terminal domain. Nm23-H2 has a structure similar to that of Nm23-H1 except for a difference in electrostatic surface potential that influences the DNA-binding properties of Nm23-H2^68^. The crystal structure of the S120G mutant, found in several aggressive neuroblastomas, showed no discernible differences compared with that of the wild type^82^.

**Fig. 1:**
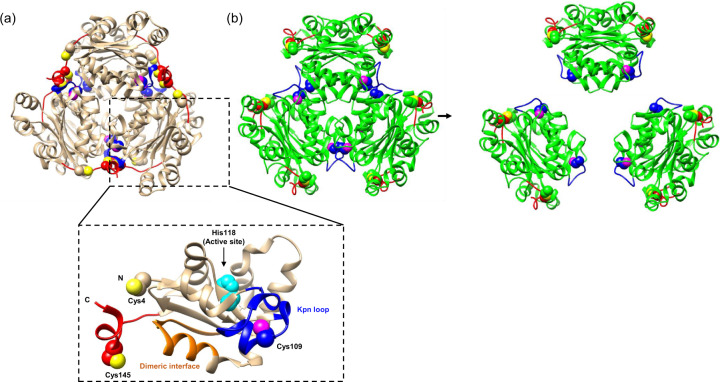
Conformational changes in Nm23-H1 under oxidative conditions^30^. The conformational change in Nm23-H1 under oxidative conditions occurs sequentially from left to right. (**a**) Superposition of a native hexamer form that has NDPK activity (colored beige). (Bottom box) The monomer form of Nm23-H1 showing key residues and domains related to oxidative conformational changes. Spheres colored yellow are sulfurs on Cys4 and Cys145, and magenta is sulfur on Cys109. The carbons of these cysteines are tan, red, and blue, respectively. Spheres colored cyan are atoms in the active site (His118). (**b**) Superposition of the oxidized hexameric form that has no NDPK activity (left, colored green) dissociates into dimers (right). The C-terminal-interacting region of the Kpn loop (aa 109–114) region is shown in blue, the C-terminal domain (aa 140–151) is shown in red, the sulfurs on Cys4 and Cys145 are yellow balls and those on the Cys109 residues are magenta balls. The carbons in those residues Cys4, Cys109, and Cys145 are green, blue, and red balls, respectively.

The oxidized crystal structure of Nm23-H1 was determined by molecular replacement using the native form (PDB entry 1jxv;90) at 2.8 Å resolution as a model^30^. Contributing to the flexibility of the C-terminus, one subunit contains 151 residues, and the other contains 149 residues, lacking one and three C-terminal amino acids, respectively. Each monomer has a core domain containing a ferredoxin-like fold and a flexible C-terminal domain (Fig. 1). The six promoters in the unit cell form a triangular structure with a bound phosphate ion in each active site. The active sites reside on the top and bottom sides of the structure. Interestingly, no prominent conformational change was detected in the active site of the oxidized form except for the distorted backbone of Gly113, influencing the binding affinity to the adenine moiety of the ADP substrate.

Some notable changes were found on residues 110–116. The effects from the flipping of the main chain conformations are clearly detected, but the positions of the side chains do not deviate much from those of the native structure. Although the shifts in side chains at Asp14, Arg105, Cys109, and Ile116 are clear, their influence on activity may be limited owing to their relatively large distance from the substrate-binding site. However, the architecture of Nm23-H1 along the equatorial surface formed by six promoters was quite different from that of the native structure. This difference was caused by the intramolecular disulfide bond between Cys4 and Cys145 under oxidative conditions. The S atoms of the two cysteines are ~20 Å apart in the native crystal structure, and the oxidation mechanism may be analogous to that of atypical 2-Cys in Prx^84^. As Cys4 was predicted to have a lower pK_a_ than Cys145 by the cysteine oxidation prediction algorithm (COPA^85^), Cys4 may be the peroxidatic cysteine, which is oxidized first. The sulfhydryl of Cys145 is then the resolving cysteine. Biochemical studies using native Nm23-H1 treated with H_2_O_2_ clearly showed that the intra-disulfide bond population increases proportionally to the H_2_O_2_ concentration, and the intra-disulfide bond was confirmed by peptide sequencing using nanoUPLC-ESI-q-TOF tandem MS with the DBond algorithm.

In the native crystal structure, the C-terminal domain of Nm23-H1 wraps around the equatorial surface and thereby stabilizes the hexameric state by interacting through two hydrogen bonds with the bottom side of the Kpn loop region of a neighboring subunit. On the other hand, the formation of an intramolecular disulfide bond under oxidative conditions induces a large conformational change in the C-terminal domain. It is triggered by the breakage of two hydrogen bonds between the C-terminal domain and the Kpn loop region. Consequently, a helix-to-loop transition occurs in the C-terminal domain, which facilitates the movement of Cys145 toward Cys4 by 20 Å. The bottom side of the Kpn loop region is thereby uncovered, exposing Cys109 to the surface where it is susceptible to oxidation toward sulfonic acid and glutathionylation^29^. The degree of the oxidative modification of Cys109 to produce sulfonic acid was measured to assess the effect of Kpn loop exposure. As expected, Cys109 of the wild type was heavily oxidized to sulfonic acid proportional to the H_2_O_2_ concentration, in contrast to the Cys109 residues of the C4S and C145S mutants that were significantly less oxidized. These results clearly indicate that intramolecular disulfide bond formation between Cys4 and Cys145 and full hexamer dissociation to form dimers likely precedes sulfonic acid formation at Cys109 (Fig. 1).

To predict the inactivation mechanism of sulfonic acid at Cys109, an energy-minimized sulfonylated Nm23-H1 model was constructed utilizing the SYBYL-X 1.3 program. The model revealed that the oxygen moiety on the sulfonyl group of Cys109 strongly attracts Arg105 and Asn115. Since the amide moiety of Asn115 is the key residue that holds the phosphate moiety of the nucleotide, the binding affinity of nucleotides to Nm23-H1 is expected to be severely diminished. As a result, NDPK activity may fully disappear, which indicates that the hexameric state of Nm23-H1 is absolutely required for its tumor metastasis suppressor activity. Studies of P96S and S120G mutants suggested that the dimeric forms with NDPK activity do not exhibit tumor metastasis suppressor activity, which indicates that some of the cellular functions of Nm23-H1 may be coupled to its oligomeric state. Three different oligomers of Nm23-H1 have been identified, namely, monomers, dimers, and hexamers, and oxidized Nm23-H1 is a dimer in the asymmetric unit. Therefore, in an alternative approach, HDX-MS was employed to observe conformational dynamics that demonstrate the transition from a hexamer into monomers under oxidative conditions. Dimeric interfaces (aa 24–40) and the Kpn loop regions (aa 94–114) of Nm23-H1 showed a large difference in HDX ratio depending on the H_2_O_2_ concentration. These regions are located at the interfaces of subunits and are blocked from solvent access in the native hexamer structure. As the concentration of H_2_O_2_ increased, these sites open to the solvent in a stepwise manner. At a low concentration of H_2_O_2_ (above 0.1 mM in our data), the Kpn loop region starts to be exposed to solvent. This phenomenon is driven by perturbation of the Kpn loop region triggered by the intramolecular disulfide bridge, as discussed above. This result is consistent with a report of P96S and S120G mutants in which perturbation of the Kpn loop region was proposed to promote dissociation from a hexamer to dimers^86,87^. At higher H_2_O_2_ concentrations (>0.5 mM), the region corresponding to the dimeric interface starts to be exposed and is accessible to the solvent, which means that the dimers can be further dissociated into monomers. The presence of a monomeric form was observed in the P96S and S120G mutants^88^. However, this monomer may be very unstable because of the hydrophobic nature of the exposed surface. In summary, Nm23-H1 hexamer is dissociated into dimers and finally into monomers with increasing H_2_O_2_ concentration. The crystal structure of oxidized Nm23-H1 provided additional insight into its molecular functions. There is almost no conformational change at the active site except for the backbone of Gly113, despite large tertiary and quaternary conformational changes. The coupling of the various molecular functions and quaternary conformations of Nm23-H1 may be an efficient way to diversify its cellular functions with its 20 or more different binding partners^89^. As the stable conformation of the Kpn loop region is important for its proper packing to form the functional hexamer, the improper confirmation of its Kpn loop region induced by oxidation or mutation seems to accelerate the dissociation of the hexamer. The behavior of the neuroblastoma-associated S120G mutant is also similar to that of the P96S mutant but is based on a different molecular mechanism. Ser120 in the core domain forms a hydrogen bond with Glu129 that also forms a hydrogen bond with the His118 active site. As Glu129 is located on the linker helix (helix H7), which mediates the anchorage of the C-terminal domain to the core domain, loss of the hydrogen bond network caused by the S120G mutant would promote flexibility of the C-terminal domain. The flexibility of the C-terminal domain would be seriously detrimental when the quaternary conformational change is initiated by certain mutations causing irreversible C-terminal flexibility, which would be exacerbated by oxidative stress^30^.

**Fig. 2:**
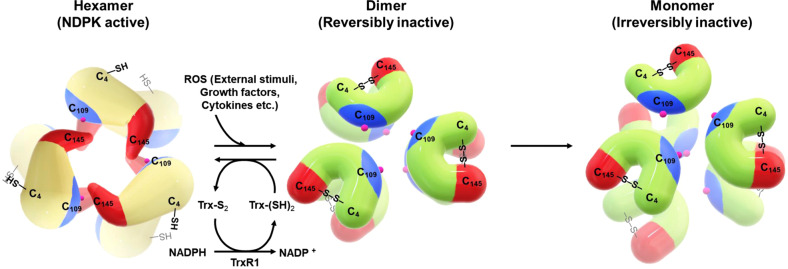
Schematic diagram of Nm23-H1 regulation based on stepwise oxidation. The colors indicate the same components depicted in Fig. 1.

In summary, Nm23-H1 is regulated by disulfide bond formation in the process of stepwise oxidative modification coupled to its oligomeric state and is easily reduced by the NADPH-TrxR1-Trx system^29^. After the first oxidation step, the hexamer Nm23-H1 dissociates into dimers triggered by intramolecular disulfide bond formation between Cys4 and Cys145. At this point, some of the cellular roles of Nm23-H1, such as its tumor metastasis suppressor activity, can be lost^71^ owing to the deformation of some of the surfaces required for binding partner proteins. However, this dimeric form can acquire other cellular roles by interacting with other partner proteins. Notably, protein phosphotransferase activity is attributed to the dimer complex of glyceraldehyde 3-phosphate dehydrogenase (GAPDH) with a dimeric state of Nm23-H1^90^. After the second oxidation step, Nm23-H1 loses its NDPK activity because of the formation of sulfonic acid at Cys109. The second oxidation is only possible when hexamer Nm23-H1 fully manifests the regulatory mechanism of Nm23-H1 under oxidative conditions (Fig. 2).

The previous study solved the puzzle to describe the regulatory mechanism of Nm23-H1 by discovering the orchestrated oxidative modifications coupled to oligomeric states through a series of experiments. This was the first study to show the regulation of the molecular and cellular functions of enzymes through stepwise oxidative modification. Because the number of examples of oxidative modifications is increasing, it may be useful to resolve complicated enzyme-regulatory mechanisms under oxidative conditions.

### Exploring a new strategy to activate Nm23-H1 as an inhibitor of metastasis

Since previous attempts to increase cellular Nm23-H1-employing gene and protein therapy or MPA treatment have some limitations in suppressing tumor metastasis, new approaches are required to increase the cellular level of Nm23 as an anti-metastatic agent. As explained previously, the NDPK activity of Nm23-H1 is conserved by maintaining the hexamer structure. It is stabilized by the interaction between the C-terminal domain and the bottom side of the Kpn loop region of a neighboring subunit via two hydrogen bonds. Quaternary structure changes in which hexamers are dissociated to dimers readily occur, and NDPK activity disappears under mild oxidative stress by the formation of intra-disulfide bonds (Cys4–Cys145), which induce a large conformational change in the C-terminal domain, making it more flexible, causing it to dissociate from the Kpn loop, and leading to the oxidation of Cys109 to sulfonic acid.

**Fig. 3:**
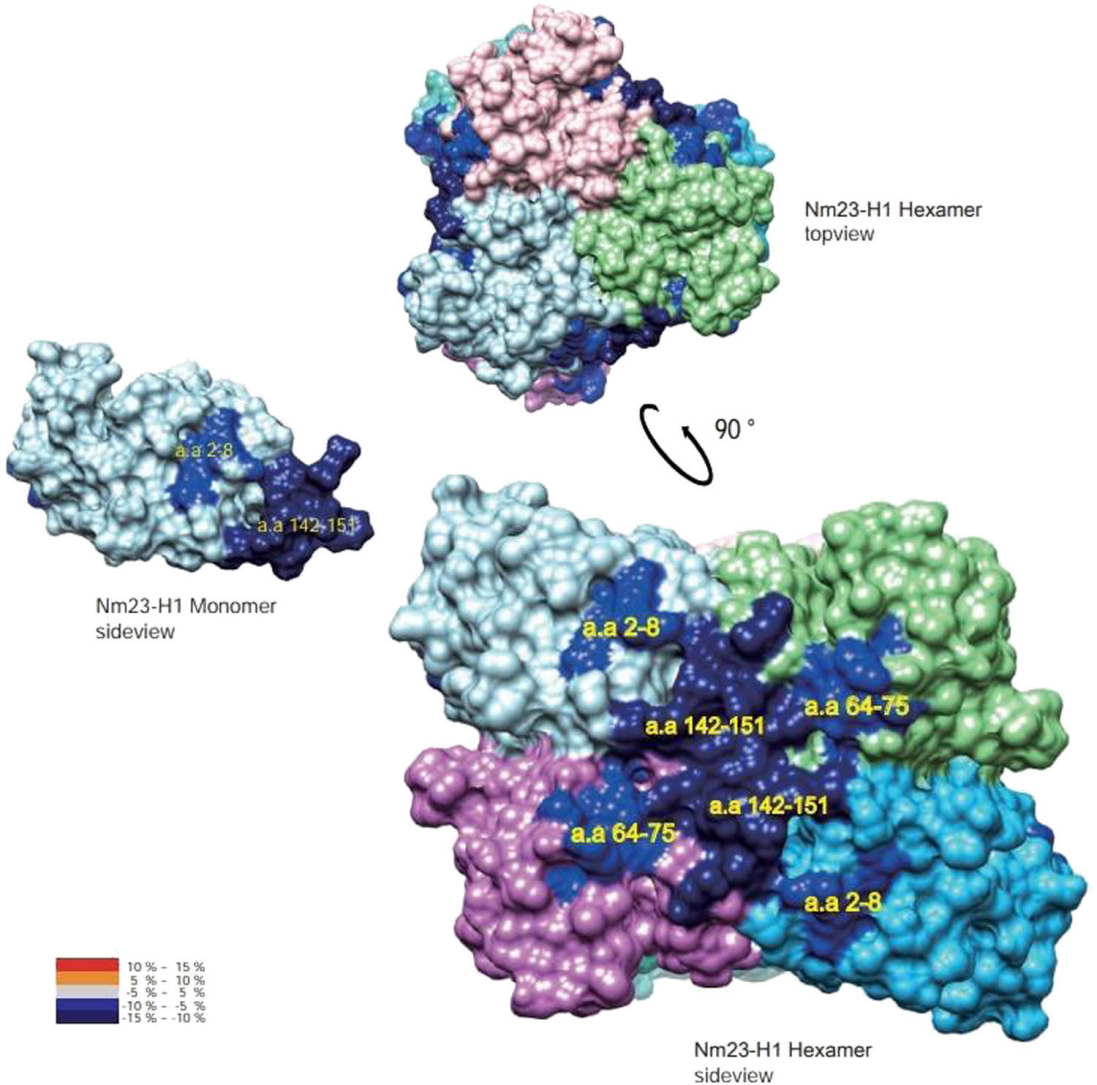
NMac1 bound to the C-terminus of the Nm23-H1 in monomer and hexamer^101^. Overlay of differential HDX data of NMac1 binding shows that NMac1 binds to C-terminal region and a peptide (aa 64–75 residue) is located in the boundary between the C-terminal region and adjacent monomer. NMac1 binding, which induces a decreasing hydrogen-deuterium exchange ratio, is presented in dark blue.

Based on structural changes under redox regulation, it is possible to propose a new strategy to find small molecules for upregulating NDPK activity by stabilizing the hexamer structure. To identify the small molecules that activate Nm23, named NMacs (Nm23 activators), compounds in a natural product library were screened upon evaluation of the increased NDPK enzymatic activity of Nm23-H1. Consequently, a small molecule, (±)-trans-3-(3,4-dimethoxyphenyl)-4-[(E)-3,4-dimethoxystyryl]cyclohex-1-ene, was found to activate Nm23-H1, thereafter called NMac1, extracted from Zingiber cassumunar rofecoxib. NMac1 directly binds to Nm23-H1 and augments its NDPK activity. Employing various NMac1 derivatives and HDX-MS, the pharmacophore and mode of action of NMac1 were identified. It binds to the C-terminus of Nm23-H1 to stabilize the hexamer structure and induces NDPK activation. To identify the binding region of NMac1 with Nm23-H1 and its mode of action, discernible changes were identified in three peptic peptides, aa 2–8, 64–75, and 142–152 residues, in a time-dependent manner by using HDX-MS^101^. Among these three peptides, hydrogen-deuterium exchanges in two peptides (aa 2–8 and 142–152) were significantly decreased by NMac1 binding, and these regions formed a small pocket in the C-terminal of Nm23-H1, indicating that NMac1 interacts with the C-terminus of Nm23-H1. In addition, these peptides are involved in the intra-disulfide linkage (Cys4–Cys145) formed by oxidation. The hexameric structure (Fig. 3) shows that the other peptide (aa 64–75 residue) is located in the boundary between the C-terminal region and adjacent monomer. In summary, NMac1 binds to the C-terminus of Nm23-H1, altering adjacent residues such as inter (aa 2–8)/intra (aa 64–75) peptides of the Nm23-H1 hexamer.

**Fig. 4:**
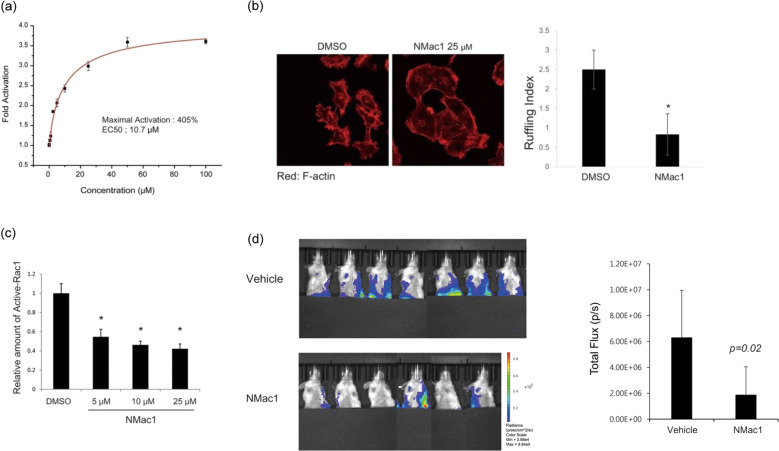
Inhibition of NMac1 on cell migration and metatstasis in triple-negative breast cancer (TNBC)^101^. (**a**) NMac1 increases the NDPK activity of recombinant human Nm23-H1 in a concentration-dependent manner. NDPK activity was measured by the amount of ATP produced from 5 ng of Nm23-H1 with 5 μM ADP & UTP as substrates in 1 min with the indicated concentrations of NMac1 or 1% DMSO as vehicle. All experiments were performed in triplicate, and the data are expressed as the means ± S.D. (**b**) NMac1 reduces membrane ruffles. (Left) Localization of F-actin was analyzed by confocal microscopy. MDA-MB-231 cells treated with 25 µM NMac1 (or 0.05% DMSO as the vehicle) for 16 h were stained with rhodamine phalloidin. (Right) The number of cell ruffles was quantified by determining the ruffling index. (**c**) NMac1 reduces Rac1 activation. An active Rac1 pull-down assay was conducted with MDA-MB-231 cells at the indicated concentrations for 16 h. A representative experiment of three independent experiments is shown. (**d**) MDA-MB-231-Luc-D3H2LN cells were orthotopically injected into NOD/SCID mice, and the mice were treated with vehicle or NMac1 (*N* = 7 for the Veh group, *N* = 6 for the NMac1 group). (Left) Bioluminescence images showing metastatic tumor cells. (Right) Quantification of the bioluminescence images 3 weeks after NMac1 treatment. The data are presented as the mean total photon flux per second ± S.D.

Because of the cooperation between the C-terminal region and Kpn loop, NMac1 binding to the C-terminus significantly upregulates NDPK activity, as shown in Fig. 4a. In addition, the morphological conversion of MDA-MB-231 mesenchymal breast cancer cells to an epithelial cell shape was observed. This morphological change, which is caused by reduced membrane ruffles, increased cell-to-cell contact and cell adhesion, and occurred via actin-cytoskeleton reorganization upon the inhibition of active Rac1-GTP formation (Fig. 4b, c). As a result, NMac1 suppressed invasion and migration in vitro and metastasis in vivo (Fig. 4d) in a breast cancer mouse model. As an activator of NDPK-A, NMac1 has potential as an anti-metastatic agent.

## Conclusion

Tumor metastasis, a major reason for the treatment failure of cancer patients, accounts for >90% of cancer deaths. However, anti-metastatic drugs are unavailable because of the complexity of related pathways in metastatic processes, although there have been a number of trials of drugs designed to inhibit metastasis. Among the trials, several attempts to increase Nm23-H1 and reduce the invasion of metastatic cancer were performed since Nm23-H1 was identified as the first MSP, regulating multiple stages in the metastasis process of breast cancer and melanoma. Various biological approaches, including AAV-mediated Nm23-H1 gene transfer, CP-Nm23-H1 transduction, and MPA-induced Nm23-H1 overexpression, were tried, and they were successful in increasing Nm23-H1 expression and suppressing cancer metastasis to varying degrees. However, these approaches have limited utilization as anti-metastatic agents. Recently, a small molecule NMac1, which activates Nm23-H1 function and overcomes the limitations of previous treatments was found to prevent breast cancer metastasis in vivo. The pharmacophore and mode of action of NMac1 on Nm23-H1 have been unveiled: this small molecule binds to the C-terminal region of Nm23-H1 and stabilizes the hexamer, which leads to the upregulation of NDPK activity. This success was possible because of the understanding of the biochemical and molecular regulation of Nm23-H1 in response to oxidative stress. Further studies on the pharmacokinetic properties and bioavailability of NMac1 may lead to the possible use of NMac1 in combination therapy with other antitumor agents for metastatic breast cancer.
